# Identification of three novel mite allergens, Der f 42, Der f 43, and Der f 44, from *Dermatophagoides farinae* by gelsolin interactome analysis^☆^

**DOI:** 10.1016/j.waojou.2025.101067

**Published:** 2025-05-27

**Authors:** Ze-Lang Cai, Shan Liu, Anyi Ji, Rongfei Zhu, Jia-Jie Chen, Kunmei Ji

**Affiliations:** a Department of Biochemistry and Molecular Biology, School of Basic Medical Sciences, Shenzhen University Medical School, Shenzhen 518060, China; b Hebei Medical University, 361 East Zhongshan Road, Shijiazhuang, Hebei Province, China; c Department of Allergy, Tongji Hospital, Tongji Medical College, Huazhong University of Science and Technology, Wuhan 430030 China; d Institute of Allergy and Clinical Immunology, Tongji Hospital, Tongji Medical College, Huazhong University of Science and Technology, Wuhan 430030 China

**Keywords:** House dust mite, Der f 42, Der f 43, Der f 44, Gelsolin interactome

## Abstract

**Background:**

House dust mites (HDMs) produce major inhaled allergens that trigger allergic diseases worldwide. The identities of the full spectrum of HDM allergenic components are not yet known. We aimed to develop a new gelsolin interactome-analysis (GIA) method for discovering and identifying novel allergens.

**Methods:**

Gelsolin-binding proteins (GBPs) in *Dermatophagoides farinae* extracts were analyzed with gelsolin-affinity resin and LC-MS/MS (liquid chromatography coupled to tandem mass spectrometry) analyses. Recombinant proteins generated from cDNAs encoding candidate allergens were expressed in a prokaryotic system. IgE binding was evaluated by ELISA (enzyme-linked immunosorbent assay), western blotting, and dot-blotting.

**Results:**

A total of 14 GBPs were assayed, including 10 known allergens and 4 candidates. Three candidates bound recombinant gelsolin, and they were named Der f 42, Der f 43, and Der f 44 by the WHO/IUIS Allergen Nomenclature Sub-committee. IgE-binding assays showed that Der f 42, Der f 43, and Der f 44 had IgE-binding rates of 7.2% (9/125), 8.5% (12/143), and 6.7% (6/90), respectively.

**Conclusion:**

GIA revealed 3 novel HDM proteins in this study and represents a new strategy for discovering and studying allergens.

## Introduction

Inhaled allergens from house dust mites (HDMs) represent a major trigger of allergic diseases worldwide. Accordingly, HDM-allergen discovery and nomenclature assignment has been a fundamental allergy research objective.^1^ Thus far, 41 groups of HDM allergens have been identified (WHO/IUIS: https://allergen.org/) with extant technologies, including predominantly allergen protein purification, cDNA library screening, and proteomic methods in combination with IgE-binding assays.^2^^,^^3^ However, the identities of the full spectrum of HDM allergenic components are not yet known.

Cytoskeletal proteins, which are expressed ubiquitously and often highly homologous across HDM species, may be important factors in IgE cross-reactivity.^4^ Among the HDM allergens, several components are related to cytoskeleton proteins. Group 10 allergen is a tropomyosin, which is involved in muscle contraction and maintenance of cell structure.^4^ Group 11 allergen, as a paramyosin, is a coiled-coil protein that forms the core of thick filaments in the muscles.^4^ Additionally, Group 16 mite antigen, a gelsolin-like molecule, not only shares key structural and functional features but also exemplifies the allergenic potential of this protein family. Der f 16 is associated with a range of allergic symptoms, including rhinitis, asthma, and atopic dermatitis.^5^

Gelsolin is a principal actin-modulating protein, interacting with actin and other cytoskeletal proteins to stabilize cytoskeletal structure.^6^ Gelsolin also binds to other proteins, such as profilin, to modulate actin polymerization. Profilin's interaction with gelsolin helps to maintain the balance of actin monomers and filaments in the cell. Utilizing gelsolin as a bait protein can enrich and identify various cytoskeleton-related proteins of HDM. However, the broader interactome landscape surrounding gelsolin-related networks in HDM remains unmapped. Systematic analysis of gelsolin interactome of HDM may reveal novel allergen components.

Here, we report a new method for discovering and identifying novel HDM allergens that employs gelsolin interactome analysis (GIA). It could compensate for the limitations of traditional allergen screening methods based solely on IgE-binding proteomics.

## Methods

The detailed information was presented in the supplemental file.

## Results

**Fig. 1:**
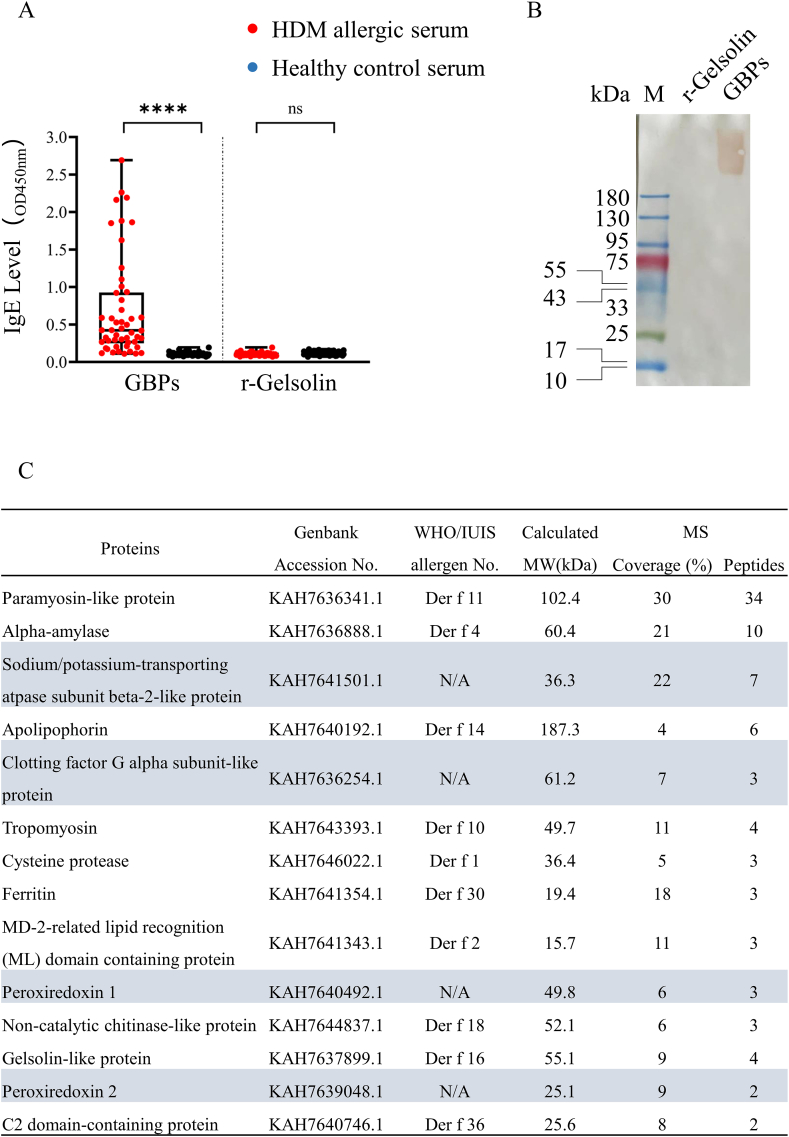
Analysis of gelsolin interactome of *D. farinae* extract A. IgE-binding activity of GBPs to 78% (39 of 50) HDM-allergic individuals' sera (vs. 50 non-allergic controls). Mann-Whitney *U* Test, n.s no significance, ∗∗∗∗p < 0.0001 vs respective control. B. Non-denaturing IgE-immunoblot of GBPs to 10 HDM-allergic individuals' pooled sera; r-gelsolin served as a control. Representative image is from 1 of 3 independent experiments. C. LC-MS/MS analysis of the gelsolin interactome; 15 proteins were selected based on the criteria of covering at least 2 peptides of the whole-protein aa sequence, with each peptide length being greater than 8 aa)

Gelsolin-binding proteins (GBPs) extracted from the common HDM species *Dermatophagoides farinae* were enriched and collected using recombinant (r-) His-tagged gelsolin affinity resin (Fig. S1A). Resin-eluted GBPs retained IgE-binding activity to serum from HDM-allergic individuals (78%, 39 of 50, *p* < 0.01) (Fig. 1A). The results were confirmed in non-denaturing IgE-immunoblotting assays (Fig. 1B). Analysis of the gelsolin interactome by liquid chromatography coupled to tandem mass spectrometry (LC-MS/MS) revealed 14 candidates whose amino acid (aa) sequences spanned at least 2 peptides at least 8 aa in length were identified, including 10 named allergens (Der f 1, 2, 4, 10, 11, 14, 16, 18, 30, and 36) and 4 novel proteins (sodium/potassium-transporting ATPase subunit beta-2-like protein (Na_K-ATPase β2), peroxiredoxin 1-like protein (Prx1), peroxiredoxin 2-like protein (Prx2), and clotting factor G alpha-subunit-like protein (CFGA) (Fig. 1C).

**Fig. 2:**
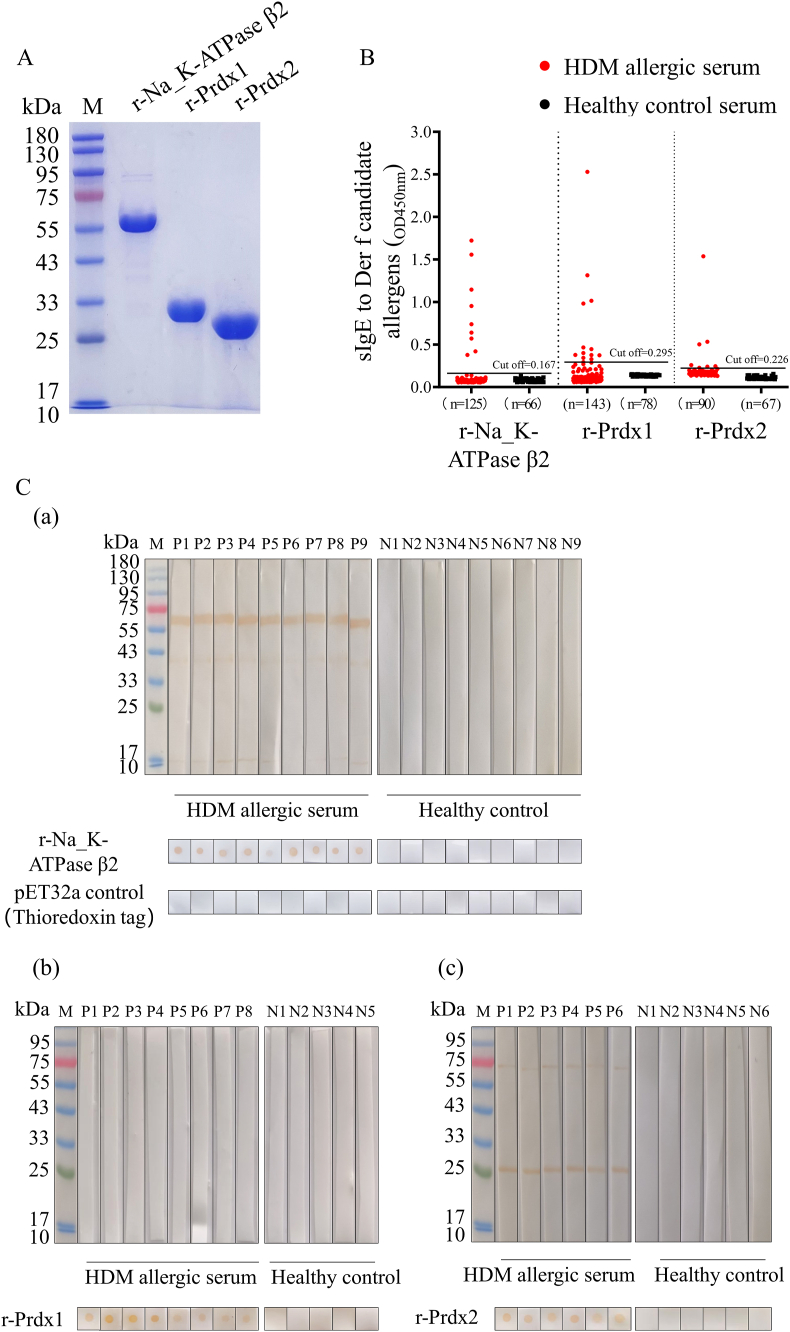
Identification of 3 novel HDM allergens from *D. farinae* A. Expression and purification of r-Na_K-ATPase β2, r-Prx1, and r-Prx2. B. IgE binding activity with r-Na_K-ATPase β2, r-Prx1, and r-Prx2 shown by IgE-ELISA with HDM allergic sera and with HC sere. Positive results in optical density require exceeding the cut-off value. The positive rate of IgE binding to r-Na_K-ATPase β2 is 9/125 (7.2%) HDM-allergic sera samples (vs. 66 non-allergic controls; cut-off value: 0.167). For r-Prx1, 12/143 (8.5%) HDM-allergic sera samples (vs. 78 non-allergic controls; cut-off value: 0. 295). For r-Prx2, 6/90 (6.7%) HDM-allergic sera samples (vs. 67 non-allergic controls; cut-off value: 0.226). C. IgE western-blot (above) and dot-blot (below) results for r-Na_K-ATPase β2 (a), r-Prx1 (b), and r-Prx2 (c) with HDM allergic patients' sera and HC sera. HDM allergic sera were confirmed to be positive by IgE-ELISAs. Representative images (A&C) are from 1 of 3 independent experiments

Next, cDNAs encoding the 4 novel *D. farinae* proteins were cloned and 3 sequences were submitted to the NCBI GenBank database (GenBank accession numbers: Na_K-ATPase β2, PQ035916.1; Prx1, PP993146.1; Prx2, PQ035917.1) (Fig. S2). The recombinant proteins were expressed in *Escherichia coli* (Fig. 2A and Fig. S3). IgE-ELISAs with HDM allergy patients' sera showed that r-Na_K-ATPase β2, r-Prx1, and r-Prx2 had IgE-binding rates of 7.2% (9/125), 8.5% (12/143), and 6.7% (6/90), respectively (Fig. 2B); r-CFGA showed no IgE-binding (0%, 0/75) (Fig. S4). Dot-blot analyses with healthy control (HC) negative-control confirmed that serum samples from HDM-allergic patients reacted with r-Na_K-ATPase β2 (9 of 9 samples), r-Prx1 (8 of 8), and r-Prx2 (6 of 6). Additionally, IgE-western blotting showed that r-Na_K-ATPase β2 (9 of 9) and r-Prx2 (6 of 6) reacted with HDM allergy patients’ serum samples, while rPrx1 did not (0 of 8) (Fig. 2B). These results suggest that Na_K-ATPase β2 and Prx2 might exist in linear epitopes for IgE binding, while Prx1 might have only conformational epitopes. LC-MS/MS showed that the 3 allergen proteins were present in *D. farinae* protein extracts (Fig. S5). Therefore, Na_K-ATPase β2, Prx1, and Prx2 were summited to the WHO/IUIS International Allergen Nomenclature Database for designation as the group allergens Der f 42, Der f 43, and Der f 44, respectively (WHO/IUIS: https://allergen.org/). Additionally, pull-down immunoassay results showed that r-Na_K-ATPase β2, r-Prx1, and r-Prx2 each binds gelsolin (Fig. S6).

## Discussion

Thus far, 41 HDM allergen groups have been characterized by traditional methods. The present work represents the first successful detection of previously undefined *D. farinae* allergen components with a newly developed LC-MC/MC-based GIA methodology, which exhibited allergen detection efficiency (93.3%, 14/15) superior to that of current techniques (eg, genomics and proteomics). The IgE-binding rates of the presently identified allergens in ELISA are suggestive of minor allergen status. Comparative analyses of Western blot versus dot blot assays suggest that Der f 42 and Der f 44 may have linear IgE-binding epitopes, while Der f 43 may have only conformational epitopes.

The discovery of minor allergens of HDM holds significant clinical implications for the diagnosis and treatment of HDM-related allergies.^7^ Clinically, the specific IgE against allergen Group 1 and 2 of HDM often accounts for about 85% of the total IgE.^8^ Ten to 20% of HDM-allergic patients still have no response to Group 1 and Group 2 allergens.^9^ Naturally, incorporating minor allergens into diagnostic tests can enhance the sensitivity and specificity of HDM allergen detection, leading to more accurate identification of allergic patients. Our study showed that the IgE binding rate of minor HDM allergens Der f 42, Der f 43, and Der f 44 was 7.2%, 8.5% and 6.7%, respectively. These findings offer a more complete understanding of the allergenic landscape of HDM. Additionally, the discovery of minor allergens may enable the development of component-resolved diagnosis (CRD) of HDM, which allows for personalized treatment plans tailored to individual allergen profiles.^10^

Gelsolin is an actin-binding protein that participates in multiple inflammatory processes and plays a crucial role in inflammation development and resolution.^11^^,^^12^ Gelsolin can also bind some cytoskeletal proteins, including the highly 3phylogenetically conserved protein myosin.^13^ Consequently, GIA can provide significant advantages in allergen detection by addressing the limitations of traditional methods and enhancing the accuracy and efficiency of identifying novel allergens from cytoskeletal proteins. Traditional proteomics screening approaches heavily rely on allergen abundance and immunological detection, often overlooking low-abundance allergens with weak IgE reactivity. GIA overcomes these challenges by utilizing gelsolin-affinity purification technology, which effectively enriches cytoskeletal proteins allergens, thereby improving the detection of clinically relevant allergens. This approach not only accelerates the identification of novel allergens but also may provide insights into the potential function of gelsolin-related proteins in developing HDM allergy.

The findings in this study are the first to suggest a potential link, but yet-to-be empirically demonstrated as a direct link, between allergenic proteins and the immunomodulatory protein gelsolin.

## Conclusion

GIA revealed 3 novel HDM proteins: Der f 42, Der f 43, and Der f 44. This method represents a new strategy for discovering and studying unknown allergens. GIA of *D. farinae* extract revealed novel allergens, which were then validated in serological immunoassay experiments, alongside known HDM allergens, which provided confirmation of methodological validity. GIA has the potential to improve the efficiencies of allergen detection and novel allergen discovery relative to currently used techniques. Further experiments are needed to explore whether gelsolin does or does not interact directly with HDM allergens.

## Data availability statement

The data that support the findings of this study are available from the corresponding author upon request.

## Author contributions

RFZ, JJC, and KMJ designed and supervised the study. ZLC, SL, and AYJ performed the experiments including purification of recombinant allergen, cDNA cloning and allergenicity assessment. ZLC analyzed the data and wrote the manuscript. RFZ, JJC, and KMJ revised it.

## Ethics approval and consent to participate

Permission to conduct this study was obtained from the Ethics Committee of the First Affiliated Hospital of Guangzhou Medical College (No. 2012-51). Informed consent was obtained from all individual participants included in the study. All procedures involving human participants were in accordance with the ethical standards of the committee.

## Authors’ consent for publication

All authors read and approved the final version of the manuscript and gave final consent for publication in WAO Journal.

## Funding information

This study was supported by 10.13039/501100001809National Natural Science Foundation of China (grant no. 82071806), 10.13039/501100003453Natural Science Foundation of Guangdong province (grant no. 2024A1515010762), and 10.13039/501100004791Shenzhen City (grant no. JCYJ20210324095004012).

## Declaration of competing interest

The authors declare that they have no relevant conflicts of interest.
