# Supplementary material for: Identification of three novel mite allergens, Der f 42, Der f 43, and Der f 44, from Dermatophagoides farinae by gelsolin interactome analysis
Source: World Allergy Organ J. 2025 May 27;18(7):101067. doi: 10.1016/j.waojou.2025.101067 (PMC12158493; doi:10.1016/j.waojou.2025.101067)
Supplement: Multimedia component 1 [file mmc1.docx]

**Supporting Information**

**Identification of three novel mite allergens, Der f 42, Der f 43, and Der f 44, from *Dermatophagoides farinae*** **by gelsolin interactome analysis**

Ze-Lang Cai, et al

**Includes：**

**MATERIALS AND METHODS;**

**SUPPLEMENTARY FIGURES:** Fig.S1 to S6;

**SUPPLEMENTARY TABLE:** Table S1;

**MATERIALS AND METHODS**

**Sera specimens**

Sera samples from 143 house dust mite (HDM) allergic patients (69 males and 74 females; age, 18–62 y) and 78 non-allergic individuals (42 males and 36 females; age, 18–68 y) were provided by the First Affiliated Hospital of Guangzhou Medical College. IgE binding specificities for HDM proteins were measured with an ImmunoCAP allergen detection system (Uppsala, Sweden); a measurement result > 4 was considered indicative of IgE specificity for the HDM allergic group.

**Expression of r-gelsolin**

The genetic locus encoding the actin-binding domain of mouse gelsolin (Ala432-Ala780, Uniprot: P13020) was synthetized with six His tags or GST tag by Genscript Biotech (Nanjing, China), and then subcloned into vector pCold II for recombinant protein expression. The protein was expressed in soluble form and purified using a Ni-NTA affinity resin (Figure S1).

**HDM extract**

*Dermatophagoides farinae* mites were cultured in an artificial climate incubator at 25 °C and 70% relative humidity in our laboratory [1]. HDM extraction was performed as described previously [1]. Protein bands were visualized with Coomassie Blue staining, and their associated concentrations were quantitated with a BCA Protein Assay Kit (Thermo, USA).

**Co-immunoprecipitation for HDM gelsolin interactome**

Gelsolin binding proteins (GBPs) in HDM extracts were enriched in co-immunoprecipitation assays. Ni-NTA affinity resin was bonded with saturated amount of recombinant (r-) gelsolin. The resin was then transferred into a new column for collecting GBPs from HDM extracts. HDM extracts from *D. farinae* passed through the column slowly overnight in 4 °C. After equilibration, GBPs were eluted with elution buffer (10 mM Tris-HCl pH 8.0, 1 mM ATP). The eluted solution was subjected to native-PAGE and IgE-immunoblot analysis.

**IgE-immunoblot analysis**

Antibody binding with purified or eluted proteins was determined by IgE western blot assays, performed as described previously [2]. Briefly, proteins were separated by native-PAGE and transferred to polyvinylidene-fluoride membranes (Millipore, USA). The blots were blocked with protein-free reagent (NYPBR01, Toyobo, Japan) to minimize masking of low signal intensities. The blots were incubated with HDM-allergic or non-HDM-allergic sera (1:5 in blocking solution) at 4 °C overnight. The blots were incubated with secondary antibodies [mouse anti-human IgE Fc-HRP (horse radish peroxidase), #9160-05; 1:2000 dilution; Southern Biotech] for 2 h at room temperature. After three washes in Tris-buffered saline with 0.1% Tween20 (TBST), protein bands were visualized with a DAB substrate kit (ThermoFisher, USA).

**IgE-ELISA analysis**

IgE-ELISAs were performed as described previously [3]. Briefly, microtiter plates were coated with indicated proteins at 4 °C. Protein aliquots were placed (200 ng/well) in highly basic (pH 9.2) carbonate buffered (0.1 mol/L) solution (Leagene, Beijing, China) overnight. The plates were blocked for 2 h at room temperature in phosphate buffered saline (PBS) with 3% bovine serum albumin (BSA). Subsequently, the sera (dilution: 1:10, PBS with 1% BSA) were incubated for 2.5 h at 37 °C. The plates were incubated with mouse anti-human IgE Fc-HRP antibody (1:2,000; Southern Biotech) for 1.5 h at 37 °C. The reactions were carried out with 100 μL of a TMB peroxidase substrate to detect bound secondary antibodies, and halted by adding 50 μL of 2 mol/L sulphuric acid. The absorbance of each well at 450 nm was measured by a microplate reader (ThermoFisher, USA). IgE-ELISA results with a positive/negative value (ratio of optical density values of positive and negative result samples) > 2.1 were considered affirmatively selective. All tests were performed in triplicate.

**HDM allergen candidates from gelsolin interactome by liquid chromatography coupled to tandem mass spectrometry (LC-MS/MS) analysis**

GBP eluate was subjected to IgE-immunoblot analysis. IgE-positive sections of the nitrocellulose membrane were cut and subjected to LC-MS/MS. The membrane was digested directly in trypsin. Data-dependent acquisition mass spectrum techniques were used to acquire tandem MS data on a ThermoFisher Q Exactive plus mass spectrometer (ThermoFisher, USA) fitted with a Nano Flex ion source. MS/MS data were analyzed for protein identiﬁcation and quantiﬁcation in PEAKS Studio 8.5. The local false discovery rate for peptide spectrum matches was 1.0% after searching against the *D. farinae* transcriptome database with a maximum of two missed cleavages. The following settings were selected: oxidation (M), acetylation (Protein N-term), deamidation (NQ), pyro-glu from E, and pyro-glu from Q for variable modifications, as well as fixed carbamidomethylation of cysteine. Precursor and fragment mass tolerance levels were set to 10 ppm and 0.05 Da, respectively (Figure 1C).

**cDNA cloning and recombinant protein expression**

Total RNA was extracted from *D. farinae* mites with Trizol/chloroform and then nanodrop quantified. cDNAs encoding sodium/potassium-transporting ATPase subunit beta-2-like protein (Na_K-ATPase β2), peroxiredoxin 1-like protein (Prx1), peroxiredoxin 2-like protein (Prx2), clotting factor G alpha-subunit-like protein (CFGA) were amplified with primers (Figure S2, Table. S1). PCR-amplified products were ligated into the expression vectors (Table S1). Each recombinant expression plasmid was translated into *Escherichia coli* BL21 (DE3) for expression. The supernatant and precipitate were collected and analyzed by SDS-PAGE. Each recombinant protein was purified by Ni-NTA gel affinity chromatography (GE Healthcare, USA) (Figure 2A and Figure S3). Protein concentrations were measured by BCA assay.

**IgE dot blot**

Nitrocellulose membranes (Millipore, USA) were cut into 0.5 × 0.5-cm squares, loaded with protein samples, air-dried, blocked with 5% BSA for 2 h at 37 °C and then incubated with the HDM allergic serum and healthy control (HC) serum samples (1:5 dilutions) for 2 h at 37 °C, separately. Mouse anti-human IgE (Fc)-HRP (SouthernBiotech, Cat. 9160-05, and 1:1000 dilution) was added for 1 h at 37 °C after washing. Binding was visualized as brown precipitates on membranes by applying DAB substrate from a kit (Thermo Scientific).

**Identification of endogenous allergen candidates by LC-MS/MS analysis**

The 25-kDa and 35-kDa *D. farinae*-extract protein-band sections of SDS-PAGE were cut out and subjected to LC-MS/MS analysis to identify putative endogenous allergen candidates (Figure S5).

**Pull‐down immuno-assays**

The genetic locus encoding the actin-binding domain of mouse gelsolin (Ala432-Ala780) was subcloned into a pGST expression vector for expressing GST-tagged gelsolin. GST-tagged gelsolin was applied to glutathione sepharose 4 fast-flow resin, and the thus functionalized resins were incubated with His-Na_K-ATPase β2, His-Prx1, or His-Prx2 at 4 °C overnight. After centrifugation, the complex pellets were washed and resuspended in denaturing Laemmli’s (SDS-Sample) buffer. Samples were run in a 12% SDS‐PAGE. The complex proteins were transferred onto nitrocellulose blots, which were blocked with TBST containing 3% BSA. Following blocking, the blots were incubated with anti-His antibodies (1:1000) at 37 °C 2 h. After three washes in TBST, the blots were incubated with a HRP-linked secondary antibodies (goat anti-mouse IgG-HRP, 1:1000 in blocking solution) for 1 h at room temperature. Proteins bands were visualized by exposure to a 1:1 ratio of chemiluminescent reagents (Meilun, Dalian, China) (Figure S6).

**Statistical analysis**

The data were analyzed and visualized in GraphPad Prism 5.0. Quantitative data are reported as means ± standard errors of the mean. Mann-Whitney U Test was employed to detect inter-group differences with significance criteria at *p* < 0.05.

**References**

[1] Chan TF, Ji KM, Yim AK, Liu XY, Zhou JW, Li RQ, Yang KY, Li J, Li M, Law PT, Wu YL, Cai ZL, Qin H, Bao Y, Leung RK, Ng PK, Zou J, Zhong XJ, Ran PX, Zhong NS, Liu ZG, Tsui SK. The draft genome, transcriptome, and microbiome of *Dermatophagoides farinae* reveal a broad spectrum of dust mite allergens. J Allergy Clin Immunol. 2015 Feb;135(2):539-48. doi: 10.1016/j.jaci.2014.09.031.

[2] Cai ZL, Liu S, Li WY, Zhou ZW, Hu WZ, Chen JJ, Ji K. Identification of an immunodominant IgE epitope of Der f 40, a novel allergen of *Dermatophagoides farinae.* World Allergy Organ J. 2023 Aug 1;16(8):100804. doi: 10.1016/j.waojou.2023.100804.

[3] Li WY, Cai ZL, Zhang BP, Chen JJ, Ji K. Identification of an immunodominant IgE epitope of Der p 39, a novel allergen of *Dermatophagoides pteronyssinus*. World Allergy Organ J. 2022 May 6;15(5):100651. doi: 10.1016/j.waojou.2022.100651.

**SUPPLEMENTARY FIGURES**

**
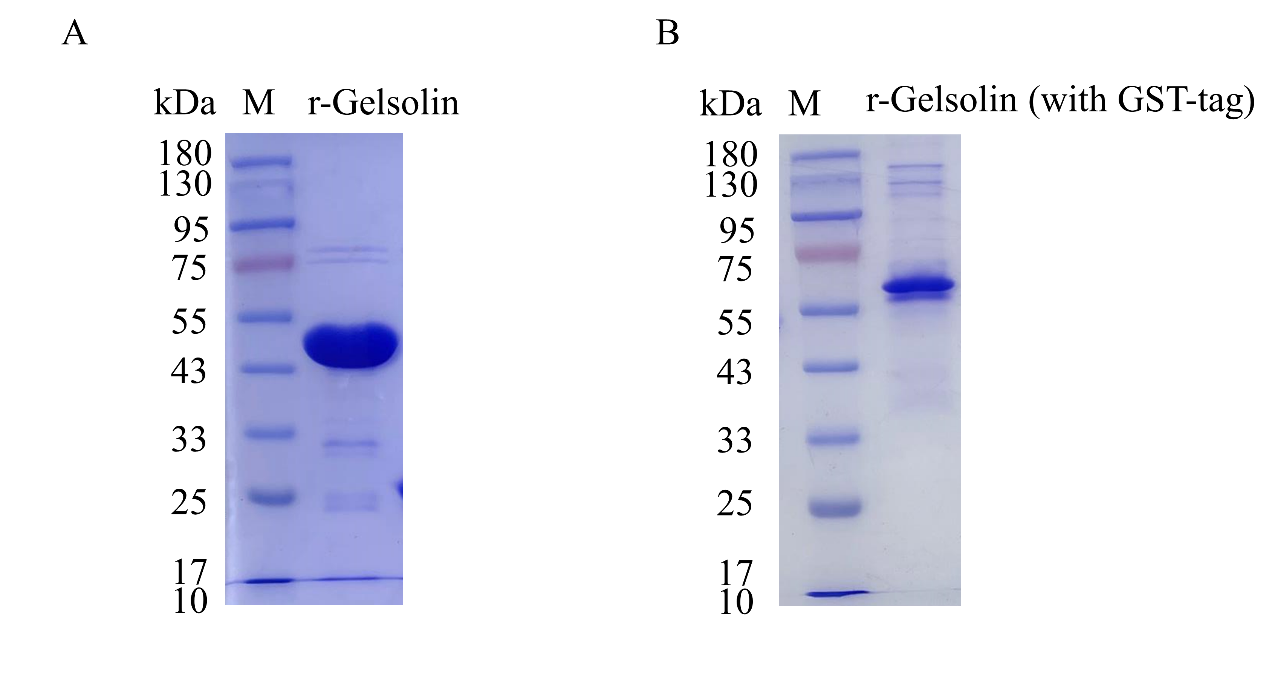
**

**Figure S1.** Expression of r-gelsolin.

SDS-PAGE of purified soluble r-gelsolin with 6× His tags (A) or GST tag (B) expressed in *E. coli*. M: pre-stained protein marker.

**
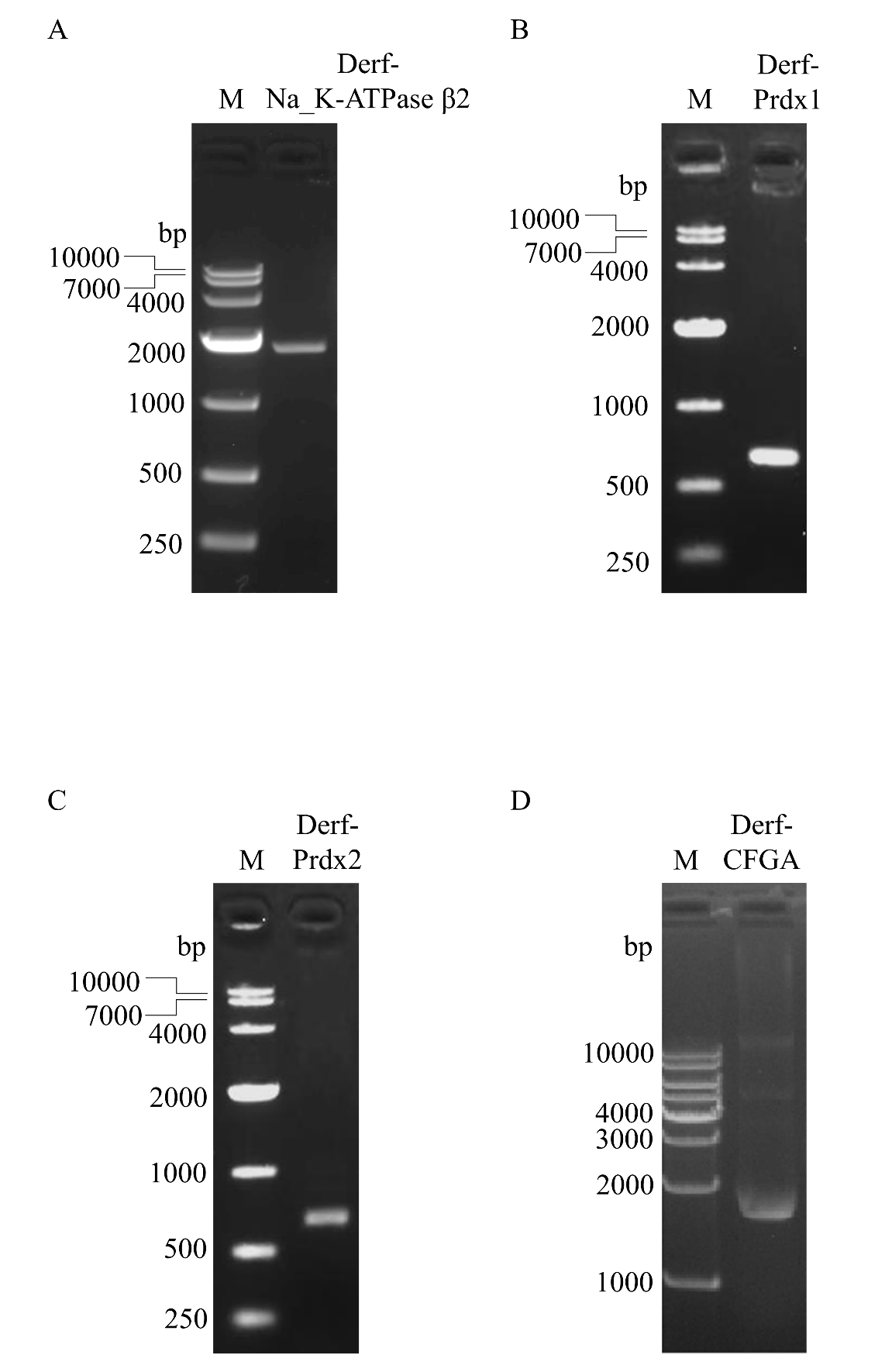
**

**Figure S2.** RT-PCR results of HDM candidate allergens.

Na_K-ATPase β2 (A), Prx1 (B), Prx2 (C), CFGA (D) open reading frame cDNAs were amplified by RT-PCR using specific primers. M: DNA Marker.

**
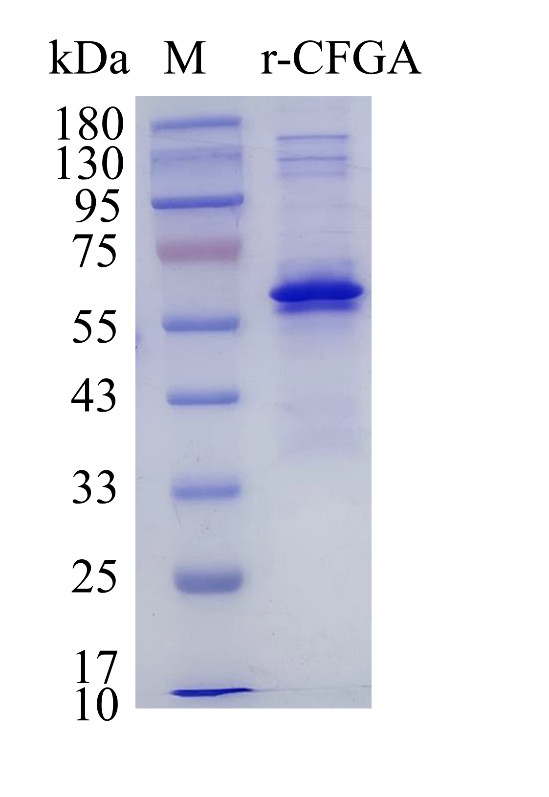
**

**Figure S3.** Expression of r-CFGA (clotting factor G alpha-subunit-like protein).

SDS-PAGE of the purified soluble r-CFGA protein with 6 × His tag expressed in *E. coli*. M: pre-stained protein marker.

**
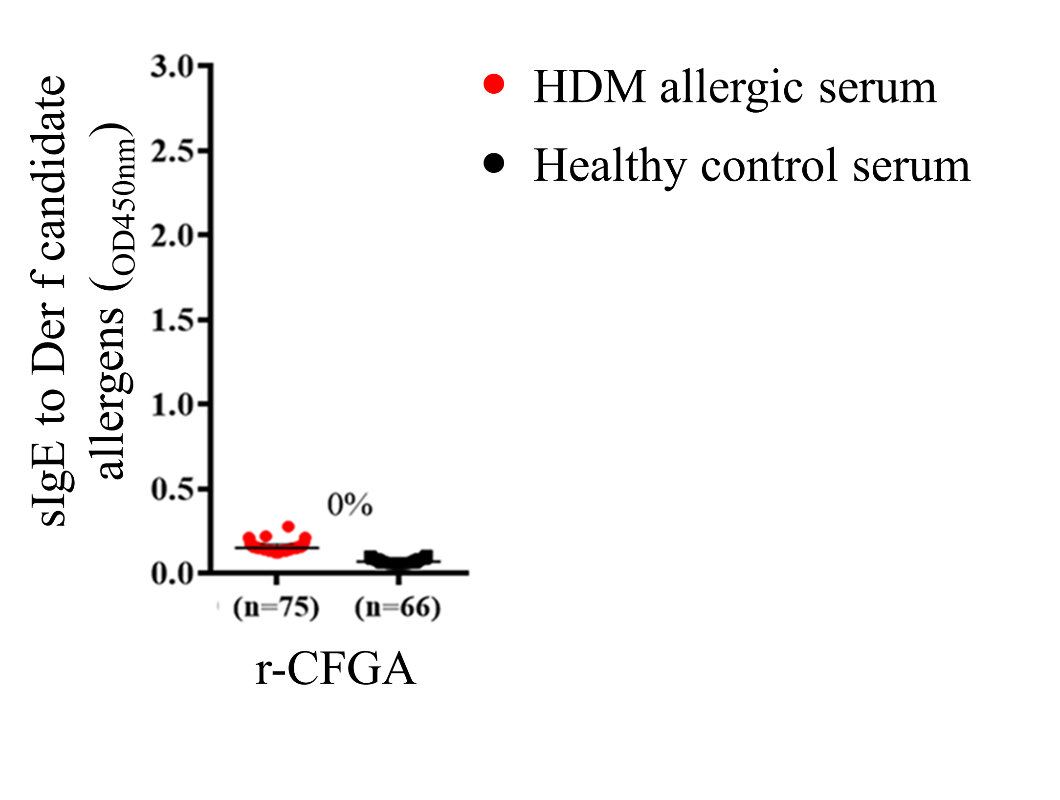
**

**Figure S4.** IgE binding capacity analysis of rCFGA.

IgE-ELISA detection of IgE binding capacity of CFGA showing no IgE-binding with HDM allergy patients’ sera.

**
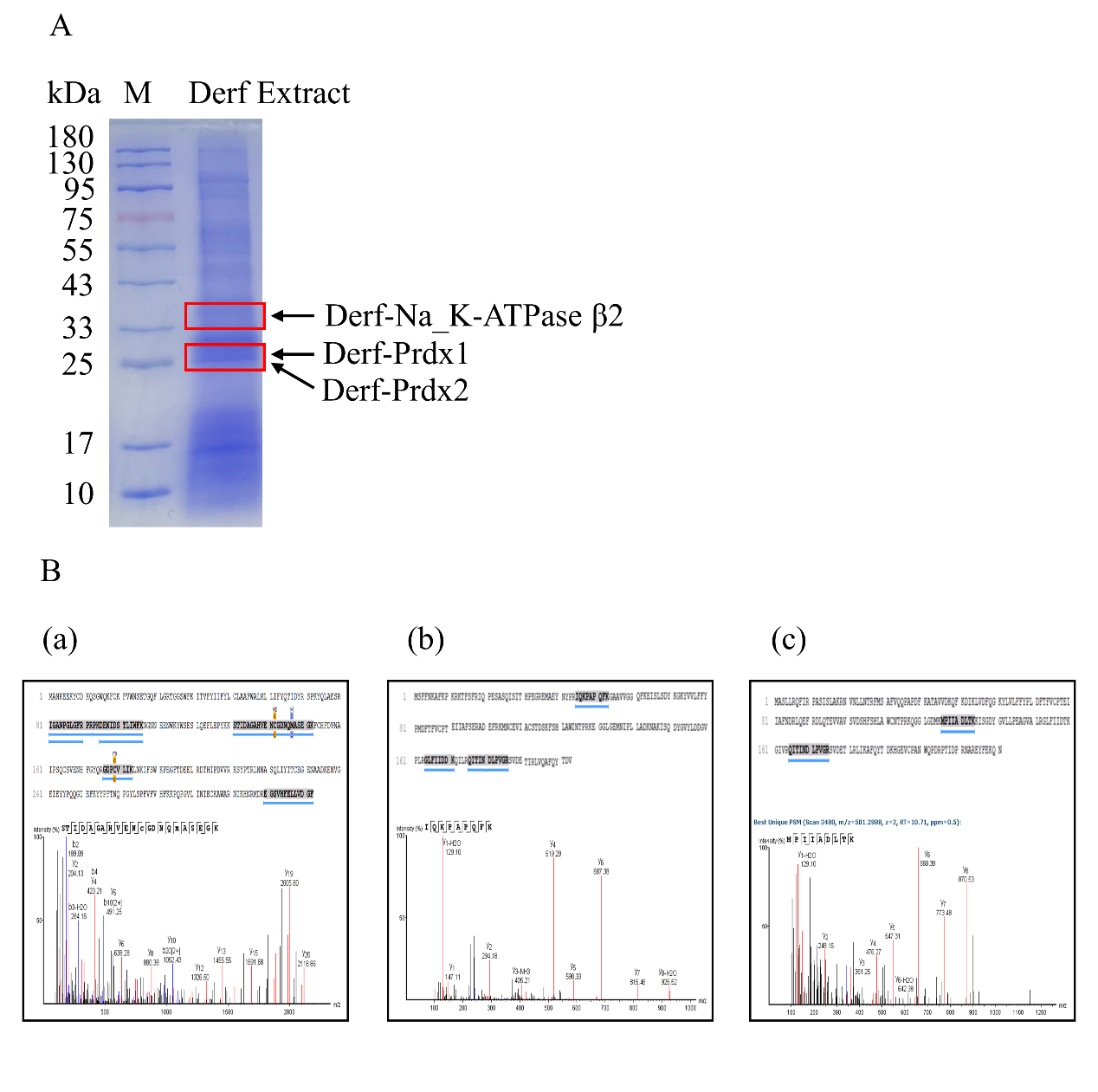
**

**Figure S5.** Identification of endogenous Na_K-ATPase β2, Prx1, and Prx2 proteins from *D. farinae* crude extract by LC/MS analysis.

A. SDS-PAGE of *D. farinae* crude extract. Natural Na_K-ATPase β2, Prx1, and Prx2 proteins may be present at 25-kDa and 35-kDa bands (red boxes). B. LC/MS analysis of the 25-kDa and 35-kDa band samples obtained from crude extract.

**
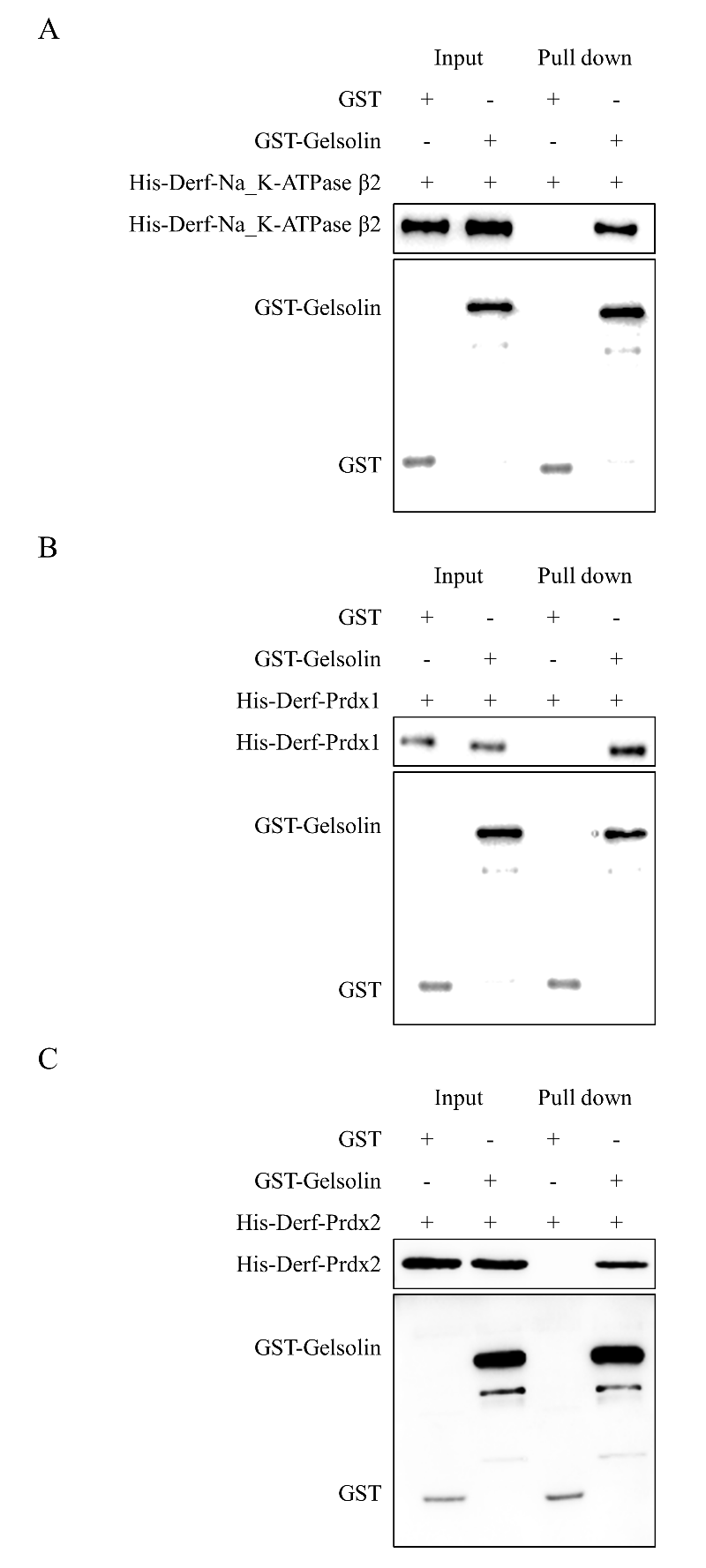
**

**Figure S6.** Pull‐down immuno-assays.

Interactions of Na_K-ATPase β2 (A), Prx1 (B), and Prx2 (C) with gelsolin were analyzed by pull-down assay.

**SUPPLEMENTARY TABLES**

**Table S1**. PCR primers and expression vectors.

|  | PCR primers | | Expression vectors |
| --- | --- | --- | --- |
| Na_K-ATPase β2 | Forward primer | 5’-ATGGCAAACAAAGAAGAAAAAT-3’ | pCold II vector  (Takara) |
|  | Reverse primer | 5’-TTAGAATCCATCGACTAATAATTCA-3’ |  |
| Prx1 | Forward primer | 5’-ATGTCATTCTTCAACAAGGCAT-3’ | pET-32a vector  (Novagen) |
|  | Reverse primer | 5’-TCAATTATTTTTGCTGAAATATTC-3’ |  |
| Prx2 | Forward primer | 5’-ATGGCATCTTTACTTCGACAATTC-3’ | pCold II vector  (Takara) |
|  | Reverse primer | 5’-CTAATTTTGTTTCTCAAAATATTCACG-3’ |  |
| CFGA | Forward primer | 5’- ATGATCGTTCAATACATTTTGAT-3’ | pCold II vector  (Takara) |
|  | Reverse primer | 5’- TTATTGCTGATAAACACGAAC-3’ |  |
